# SecMDGM: Federated Learning Security Mechanism Based on Multi−Dimensional Auctions

**DOI:** 10.3390/s22239434

**Published:** 2022-12-02

**Authors:** Qian Chen, Lin Yao, Xuan Wang, Zoe Lin Jiang, Yulin Wu, Tianzi Ma

**Affiliations:** 1 School of Computer Science and Technology, Harbin Institute of Technology Shenzhen, Shenzhen 518000, China; 2 Peng Cheng Laboratory, Shenzhen 518000, China; 3 Guangdong Provincial Key Laboratory of Novel Security Intelligence Technologies, Shenzhen 518000, China; 4 School of Software, Harbin Institute of Technology, Harbin 150000, China

**Keywords:** game theory, federated learning, mechanism design, auction theory, partial homomorphic encryption

## Abstract

As a newly emerging distributed machine learning technology, federated learning has unique advantages in the era of big data. We explore how to motivate participants to experience auctions more actively and safely. It is also essential to ensure that the final participant who wins the right to participate can guarantee relatively high−quality data or computational performance. Therefore, a secure, necessary and effective mechanism is needed through strict theoretical proof and experimental verification. The traditional auction theory is mainly oriented to price, not giving quality issues as much consideration. Hence, it is challenging to discover the optimal mechanism and solve the privacy problem when considering multi−dimensional auctions. Therefore, we (1) propose a multi−dimensional information security mechanism, (2) propose an optimal mechanism that satisfies the Pareto optimality and incentive compatibility named the SecMDGM and (3) verify that for the aggregation model based on vertical data, this mechanism can improve the performance by 2.73 times compared to that of random selection. These are all important, and they complement each other instead of being independent or in tandem. Due to security issues, it can be ensured that the optimal multi−dimensional auction has practical significance and can be used in verification experiments.

## 1. Introduction

With the deep integration and application of artificial intelligence in different industries [1,2,3] and social life [4,5,6], model training needs more participants. In this paper, we discuss how to motivate participants to provide their multi−dimensional information, such as data [7,8] and computing power.

However, data privacy and security risks [9,10] have caused widespread concern and even anxiety and panic. Federated learning [11,12,13,14] is attracting increasing attention as it can build aggregation models by ensuring that there is no need to submit raw data outside the local area [15,16,17]. Federated learning plays a perfect role in protecting privacy in model aggregation [18,19], but a suitable mechanism is needed to motivate someone to participate in this task before computing. Zhan et al. [20,21] focused on how to motivate clients effectively to participate in federated learning reliably and conducted extensive research on the existing work about incentive mechanisms for federated learning. From an economic perspective, federated learning only provides a technology but does not consider why the clients should participate or what benefits they can gain. However, Zhan et al. did not consider security and multi−dimensional information in the mechanism. Therefore, we need to provide a better mechanism for federated learning.

We need a standard and unified framework and theory with which we can compare and research the advantages and disadvantages of mechanisms [22,23] in the resource allocation and system design of federated learning. This is why we thought of the theory of auction mechanism design [24,25,26]. A suitable mechanism allows the game decision to obtain better payoffs, ceteris paribus, and it is applied in many fields [6,27,28]. In the latest edition of Nature, DeepMind [7] discusses human−centered mechanism design options. The design of the mechanism is present in almost all resource allocation issues, including taxation, voting, and spectrum auctions.

The three critical mechanism design theories are auction, contract, and bargaining. In the 1960s, William Vickrey [29] and Armando Ortega Reichert made pioneering contributions to auction theory. The 2020 Nobel Prize in economics was awarded to Paul Milgrom for his contributions to improving auction theory and new auction forms. As he said, when a traditional economic theory cannot explain and calculate the complex scenes of reality, we require a high−performance computing solution based on traditional theory to evaluate the mechanisms, getting better social welfare in the meanwhile. Therefore, it is ideal to explore the challenges of federated learning by mechanism design.

The most well−known application of auction theory, on which this paper is based, includes advertising positions [30,31] and antique auctions. However, all problems related to resource allocation are inseparable from auction theory. This is a completely theoretical system that goes far beyond the auction application. First−price auction is a simple process [32] in which many buyers bid on an item and the highest bidder procures the item (paying the highest bidding price).

Homomorphic encryption is a unique method supporting algebraic operations in ciphertext data. It plays a central role in the security mechanism discussed in this paper. On the Internet, clients in federated learning need to not only compete for participation but also ensure the quality and security of the winner. Therefore, it is necessary to develop an improved auction mechanism. It is critical to ensure that the bidding information is not intercepted by competitors or leaked and that the clients submit real multi−dimensional information. Hence, a secure multi−dimensional optimal auction mechanism is crucial.

The ubiquitous online advertising [33,34] auction faces a similar security problem as federated learning. This is just a snapshot of many scenes we have not explored yet, and the mechanical design of federated learning discussed in this paper is one of the most representative. As it considers not only the security during the auction but also the quality after the auction, it requires studying a multi−dimensional mechanism. Traditional auctions are price−oriented, and considering only price will lead to sacrificing quality. However, when participants are requested to provide information other than price, such as multi−dimensional information about their counting ability, this can compromise their privacy. This seems to be a contradiction. Therefore, we need to explore a secure auction mechanism for federated learning that can guarantee the quality of participants and the privacy of multi−dimensional information. We solve this problem for the first time by designing security mechanisms named the SecMDGM for multi−dimensional information in federated learning.

The optimal mechanism is for participants to voluntarily provide information regarding their actual price and computing power and other multi−dimensional information. An auction with this feature is called “strategy−proof,” and the auction mechanism that follows this winner−pick rule is called the “direct mechanism.” The idea of strategy−proof as the core of auction design was first given by the American Nobel Prize winner William Vickery. However, does this still hold for the case of multi−dimensional encrypted messages? Designing a mechanism to maximize the expected profit and satisfy the Pareto equilibrium and incentive compatibility is necessary, which is addressed in Section 3.

**Figure 1:**
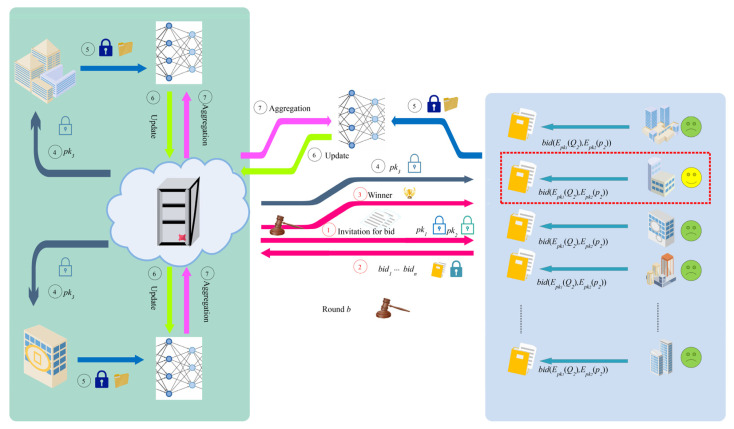
Framework for the design of federated learning security mechanisms.

Figure 1 provides the framework of the SecMDGM mechanism, including the auction stage (select the participant) and the calculation stage (agglomerate the model) of federation learning. Unlike other research on federated learning, we consider both quality and security for the first time. This is why we do not compare it with other papers in an experiment in Section 4. Briefly, we focus more on the right side in Figure 1. This paper is an extension of federated learning.

This paper explores how to make different participants more active in federated learning upfront and ensure that the final winner can provide a higher−quality model instead of a low price. Meanwhile, focuses on the privacy protection of personal multi−dimensional data. To sum up, the contributions of this paper are as follows:

(1) Security:
  (1) We realized the computation of the ciphertext score function for mechanism design based on partial homomorphic.
  (2) We redeployed the federated learning process to cover bid submission, auction calculation, and model aggregation security.
(2) Necessity:
  (1) The mechanism proposed in this paper considers the multi−dimensional information of bidders, which is more in line with actual demand.
  (2) It is proved that our mechanism is Pareto optimal and satisfies incentive compatibility (IC), which is the primary measure of mechanism performance.
  (3) It is proved that the mechanism can maximize the participants’ profit, and the equation and proof of the optimal strategy are provided.
(3) Effectiveness:
  (1) Experiments show that our mechanism can improve the accuracy of the federated learning model while ensuring security.
  (2) Our mechanism is also suitable for vertical data.

The rest of this paper is organized as follows: Section 2 presents the work on mechanism design, federated learning, and cryptographic algorithms and the relationship between them. Section 3 theoretically proves the security and necessity of the proposed multi−dimensional security mechanism. In Section 4, the effectiveness of the proposed mechanism is experimentally verified. Section 5 provides the conclusion.

## 2. Preliminaries and Notations

This paper explores the secure federated learning mechanism under multi−dimensional information for the first time from the game theory perspective. Not only does it guarantee the feasibility of security in multi−dimensional auctions, it also proves that the multi−dimensional mechanism can obtain a dominant strategy in the cryptographic process and satisfies incentive compatibility, which also enables participants to obtain the maximum expected revenue.

Since many fields of knowledge are involved here, Section 2.1 introduces auction theory in mechanism design and the difficulty of multi−attribute auctions, Section 2.2 discusses partial homomorphic encryption and its security guarantees, and Section 2.3 discusses the vertical federation learning model. 

They are interdependent, and multi−dimensional auctions can only provide the optimal mechanisms in theory. They cannot guarantee the feasibility of the application because participants will be reluctant to submit crucial information for fear of their privacy being compromised, even if they know that this is the optimal strategy. Partial homomorphic encryption solves this problem. As a new distributed solution, computing security in federated learning has also been deeply explored. However, in addition to the security of the calculation itself, security is necessary for the participant selection stage as well, especially when the participants are providing multi−dimensional private information instead of simply price. High−quality participants decide the performance of the final aggregation model. 

### 2.1. Multi−Dimensional Auction and Mechanism Design

In this paper, the design of the federated learning mechanism is the core issue. Even the discussion about security and quality is based on the mechanism design itself. Therefore, the most crucial thing is comprehending the mechanism design theory and the relationship between mechanism design and auction theory in federated learning.

**Figure 2:**
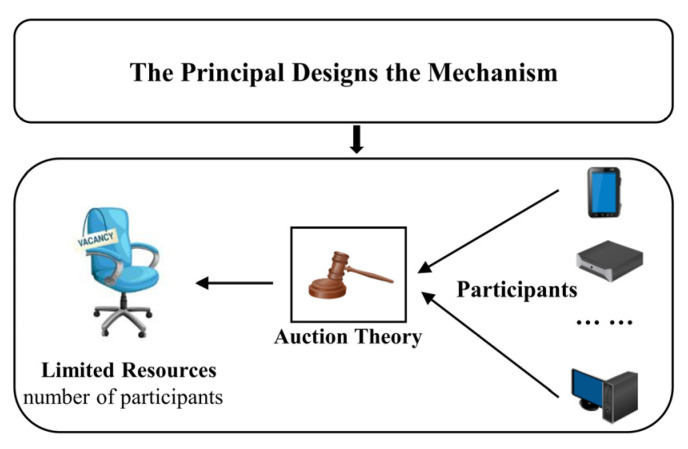
The relationship between mechanism design and auction theory in federated learning.

The principal−agent model is the basic analytical framework of mechanism design theory, as shown in Figure 2. First, the principal designs a mechanism, which is the main contribution of this paper. Second, multiple participants compete to participate in federated learning according to the given auction mechanism. Auction, bargaining, and cooperation theory are the three major mechanism design theories. An auction is a market mechanism in which participants make bids and determine the allocation of resources and the price paid according to a set of defined rules [35]. Paul Milgrom was awarded the Nobel Prize in Economics for his important contributions to auction theory.

We abstract the limited participating resources on the server as the auction object of the mechanism, and the participants who want to participate in the model aggregation become buyers. Under the optimal mechanism, the service provider can maximize social welfare by selecting the best participant, which can be the best performance of the focusing function. Each participant can honestly provide the highest−quality bidding information for maximum benefit. Under the optimal mechanism, the service provider can improve the performance of the aggregation function by selecting the right participants to maximize social welfare.

**Figure 3:**
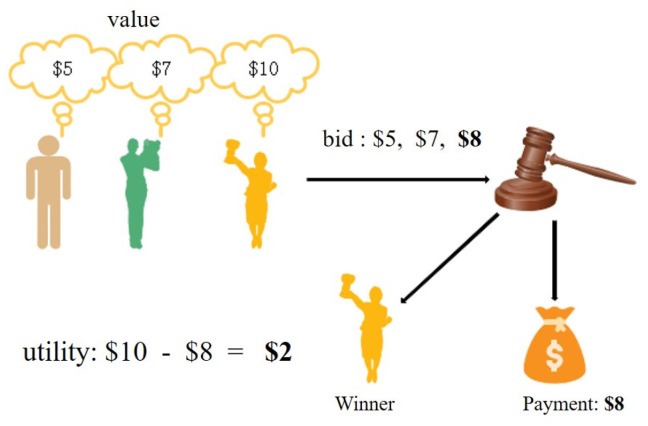
First−price auction of a single dimension.

Mechanism design theory provides ways to avoid these dilemmas under specific circumstances. Dominant mechanisms allow participants to show their personal preferences and achieve social goals. To better understand the auction mechanism, we list examples of a first−price auction. As shown in Figure 3, in the first−price auction, each participant uses a specific value to bid for the item [36,37]. At this time, the auction mechanism must make two decisions. First, who will obtain the item? It can be seen that the third participant obtained the item, with the highest bid price. Second, what amount needs to be paid? The winner needs to pay USD 8. This is one of the most manageable single−dimensional auctions that can help us understand the multi−dimensional auction theory better.

In the traditional mechanism design, the VCG auction mechanism satisfies the conditions of incentive compatibility (IC) and individual rationality (IR), which can maximize the expected profit of participants. This is effective in single−item single−dimensional auctions, but single−item multi−attribute auctions require further exploration.

**Definition** **1.** *VCG Mechanism.*

The allocation x(b) and payment rules p(b) of the VCG mechanism satisfy the following two equations, respectively:

(1) $$x_{i}\left(b\right)=\underset{\omega \in \Omega }{argmax}\overset{n}{\underset{i=1}{\sum }}b_{i}\left(\omega \right)$$

(2) $$p_{i}\left(b\right)=\left(\underset{\omega \in \Omega }{max}\underset{j\neq i}{\sum }b_{j}\left(\omega \right)\right)-\underset{j\neq i}{\sum }b_{j}\left(\omega^{*}\right)$$

where *b* stands for the bid of the participants

Here, is an alternative formula representation of the payment rules in the VCG mechanism.

(3) $$p_{i}\left(b\right)=b_{i}\left(\omega^{*}\right)-\left[\overset{n}{\underset{j=1}{\sum }}b_{j}\left(\omega^{*}\right)-\underset{\omega \in \Omega }{max}\underset{j\neq i}{\sum }b_{j}\left(\omega \right)\right]$$

This can help us better understand the theoretical proof of the SecMDGM mechanism in Section 3. The multi−dimensional auction originated from the requirements of the U.S. Department of Defense for the procurement and supply of weapons. Competitive procurement of weapons considers not only price but also various performance metrics of the weapon, including technical characteristics, delivery dates, labor performance, and estimated program costs. Equation (4) provides a simple score function for measuring the mechanism design, which is related to the utility function and price *p* induced by the multi−dimensional feature information $Q=\left(q_{1},q_{2},\cdots ,q_{m}\right)$.

(4) $$Score=U\left(q_{1},\cdots ,q_{m}\right)-p$$

Bichler defined multi−dimensional auction [38] as an auction mode in which multiple attributes of the item are considered when buyers and sellers trade, that is, an auction mode in which the two parties conduct multiple negotiations on other quality attributes in addition to the price. The experimental research shows that, for buyers, the multi−dimensional auction is better than the single−dimensional auction. Sometimes, the situation of suppliers will also improve. However, we improve the multi−dimensional auction theory via practical applications in federated learning and avoid the leakage of private information by guaranteeing security.

Many practical and theoretical problems, such as the formulation of laws and regulations, administrative management, and democratic elections, can be transformed into mechanism design problems. This paper translates the federated learning problem into an optimization problem for the auction mechanism, and the proof and measurement index of the SecMDGM mechanism is provided in Section 3.

### 2.2. Partial Homomorphic Encryption

Compared with the single−dimensional auction, the multi−dimensional auction can improve the quality of the participants but requires providing more personal information. From a theoretical point of view, multi−attribute auctions are enough. However, from a practical point of view, we need to design a multi−attribute auction mechanism and ensure the security of the bidding information. Therefore, the SecMDGM mechanism needs the support of cryptography. 

Homomorphic encryption [39] can support algebraic operations of ciphertext. To satisfy the requirements of confidentiality, cryptographers have designed different types of encryption methods according to different NP−hard problems. According to the different computing powers, these can be divided into partial and fully homomorphic encryption. Unlike the slower computation of fully homomorphic encryption [33,34,35], partial homomorphic encryption [40] is compatible with our auction mechanism in terms of security and computation performance. This is the first time that the security method and the multi−dimensional mechanism have been skillfully combined and provide strict theoretical proof.

The Paillier encryption algorithm is a representative additive homomorphic algorithm invented by Paillier in 1999 [41], based on the difficult question in the compound residual category. Moreover, the RSA encryption algorithm is the first that can be used for encryption and digital signatures that are easy to understand and operate. After surviving various attacks, it has gradually become widely accepted. It is generally considered one of the best public key cryptography algorithms and representative of multiplicative homomorphism algorithms. The SecMDGM mechanism skillfully uses the characteristics of Paillier and RSA encryption algorithms and applies them to the auction mechanism.

In this paper, fully homomorphic encryption is not considered because (1) it will be costly in terms of time and (2) the server is completely trustworthy in federated learning. Security can be achieved by adding several encryption and decryption calculations. Therefore, because the time cost of a partially homomorphic encryption algorithm can be completely ignored, this paper does not make an experimental comparison of time complexity.

In this paper, partial homomorphic encryption provides theoretical support for security calculation. A public−key cryptosystem, for all keys (*pk*, *sk*), that is generated by the key generation algorithm *Gen*(1*^n^*), has a plaintext space *M* and a ciphertext space *C*. For messages $m_{1},m_{2}\in M$ in plaintext space, the corresponding ciphertext data $c_{1}=\;E_{pk}\left(m_{1}\right)$ and $c_{2}=\;E_{pk}\left(m_{2}\right)$ satisfy Equation (5).

(5) $$\begin{array}{c} Dec_{sk}\left(c_{1}\circ c_{2}\right)\;=\;m_{1}\circ m_{2} \end{array}$$

If the operator ∘ is an additive operation, homomorphic encryption is called additive homomorphic. If it is a multiplicative operation, it is called multiplicative homomorphic. If an encryption method is homomorphic to only one operation, it is called partial homomorphic encryption. Otherwise, it is called fully homomorphic encryption. We prove the security of the encryption algorithms of Paillier and RSA as follows.

#### 2.2.1. The Security of Paillier Additive Homomorphic Encryption

The encryption and decryption process of Paillier, where *pk_2_* = (*n*, *g*) is the public key and *sk_2_* = (λ, μ) is the private key, is as follows:

First, the key needs to be generated.

(1) Randomly choose two large prime numbers *p* and *q* that satisfy *gcd*(*pq*, (*q* − 1)) = 1 and satisfy *p* and *q* of equal length.
(2) Calculate *n* = *pq* as well as λ=lcm(p − 1,q−1). Here, *lcm* denotes the least common multiple.
(3) Randomly select integer $g\in Z_{n^{2}}^{*}$.
(4) Define the function $L\left(x\right)=\frac{x-1}{n}$ and calculate $\mu ={\left(L(g^{\lambda }modn^{2})\right)}^{-1}\mathrm{mod}n$.

Here, is Paillier’s encryption process.

(1) Input a plaintext *m* that satisfies 0≤m≤n.
(2) Choose a random number *r* that satisfies 0≤r<n and $r\in Z_{n}^{*}$.
(3) Calculate the ciphertext $c=g^{m}r^{n}mod\;n^{2}.$

Paillier’s decryption process is as follows:

(1) Input a ciphertext message *c* that satisfies $c\in Z_{n^{2}}^{*}$.
(2) Calculate the plaintext $m=L\left(c^{\lambda }mod\;n^{2}\right)*\mu \mathrm{mod}n.$

Paillier satisfies the standard definition of security for encryption schemes, semantic security, i.e., indistinguishability under chosen−plaintext attack (IND−CPA). Intuitively, the ciphertext does not reveal any information in plaintext. The security of Paillier can be reduced to the decisional composite residuosity assumption (DCRA), i.e., given a composite number *n* and an integer *z*. It is NP−hard to determine whether *z* is *n* times residual under mod *n*^2^, i.e., whether there exists *y* satisfying $z\equiv y^{n}\left(mod\;n^{2}\right)$.

#### 2.2.2. The Security of RSA Multiplicative Homomorphic Encryption

Assuming that the ciphertext is c and the plaintext is m, the encryption process of RSA is as follows:

(6) $$c=m^{E}\mathrm{mod}N$$

where *pk_1_* = (*E*, *N*) is the public key and *sk_1_* = (*D*, *N*) is the private key. Here, we use the public key to encrypt and the private key to decrypt. When *N* = p*q, *L* = *lcm*(*p* − 1, *q* − 2), and *gcd*(*E*, *L*) = 1 are satisfied when 1 < *E* < *L* and E*D mod *L* = 1 when 1 < *D* < L, the key is generated.

The decryption process of RSA is as follows:

(7) $$m=c^{D}\mathrm{mod}N$$

The security of the RSA encryption scheme depends on whether the attacker can quickly obtain the plaintext *m*. The easy method is to obtain the plaintext *m* by finding the private key *D*. To obtain the private key *D*, we need to decompose *N* into *p* and *q* and then calculate *p*−1 and *q*−1 to derive *D* from *E*. Thus, the security of RSA relies on the knowledge of number theory that factorizing a large integer’s prime factor is difficult and inefficient. Briefly, it relies on the difficulty of factoring large numbers. There is no polynomial−time method for factoring the decomposition of large factors. 

Figure 1 shows the overall framework of this article. The secure computing of federated learning refers to steps 4 to 7 with *pk_3_*, which experts have thoroughly studied. Our research focuses on steps 1 to 3, with *pk_1_* and *pk_2_* corresponding to RSA and Paillier, which are not in traditional federated learning. With these steps, clients can participate more actively in model aggregation. The improvement of the multi−dimensional auction has further improved the quality of the winner. In addition, the guarantee of partial homomorphic encryption ensures that participants are no longer worried about data leakage. In Section 3, we directly use *Encrypt/E*() and *Decrypt/D*() to represent the encryption and decryption functions.

### 2.3. Vertical Federated Learning

The Google team [42] initially proposed federated learning to explore a predictive model that enables multiple smartphones to learn and share cooperatively. Different from traditional distributed learning, it can aggregate models without taking the original parameters out of the local area. The key Equation (8) is as follows:

(8) $$\omega \left(t+1\right)=\frac{\sum_{i=1}^{N}C_{i}\omega_{i}\left(t+1\right)}{\sum_{i=1}^{N}C_{i}}$$

where *C_i_* is the weight of the participants

One of the important issues unsolved in federated learning is the non−independent homogeneous distribution of data. The SecMDGM mechanism of this paper involves this problem because it measures multi−dimensional information and more dimensional information can provide a higher−quality winner for the server.

Traditional horizontal federated learning considers that two datasets have more overlapping features and fewer overlapping users. However, in real situations, the data providers in horizontal federated learning often compete.

**Figure 4:**
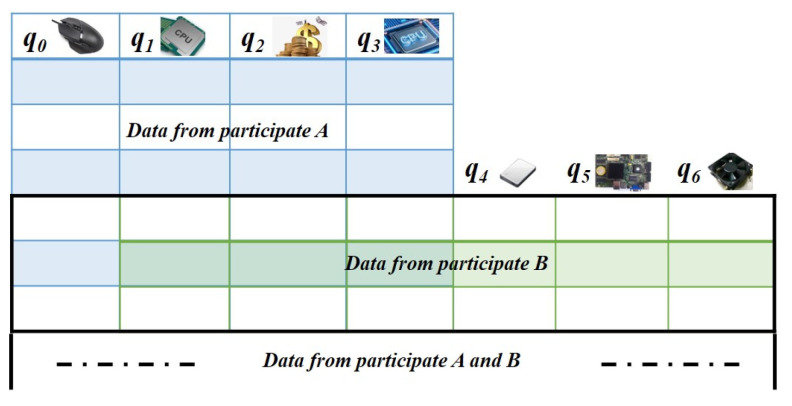
Vertical federated learning of multi−dimensional data.

As shown in Figure 4, each column represents the respective features of the participants. Since the features of the participants are different, it does not satisfy an independent homogeneous distribution. However, vertical federated learning that considers multi−dimensional data can improve the model’s accuracy.

Therefore, we design an incentive mechanism based on vertical data. Vertical federated learning is considered when there are more users overlapping and fewer features overlapping. This is consistent with our idea of the multi−dimensional information auction, which not only improves the performance of the server model but also performs experimental validation from the perspective of vertical federation learning in Section 4.

## 3. Theory of SecMDGM Mechanism for Federated Learning

As a distributed integration approach, federated learning has many advantages. How to make clients voluntarily submit higher−quality data or local computing performance is an important area of focus. We design an incentive mechanism named SecMDGM to facilitate federated learning tasks based on the theory of first−price auction. The use of multi−attribute auctions is intuitive, but it is essential to completely prove the theory since this allows us to do away with the theoretical system of the VCG model, given in definition 1. We need to improve the proof to ensure that such a mechanism is feasible and necessary. Since participants provide more and more private information, how can they reduce their worries about the information being leaked, even if they can theoretically get the best benefits? Moreover, whether the participants of multi−round federated learning will steal bid information from each other is also a security issue. 

The multi−dimensional bid information of the auction is calculated in the score function, which is mentioned in Equation (4). It is important to ensure the security of the score function, which also protects the safety of the client’s original private information. Due to the particularity of the federated learning task, the server is trusted by default, so participants only get the public key. Only the server has the corresponding private key.

This section of the paper looks at how to ensure that the score function of the server is not leaked, a security guarantee that needs to be achieved to ensure that the multi−dimensional bidding information of clients will not be leaked. In addition, this section will theoretically prove the necessity of the SecMDGM mechanism from the perspectives of Pareto optimality and incentive compatibility. This means that the mechanism can maximize the revenue of all parties. 

### 3.1. Algorithms and Framework of the SecMDGM Mechanism

The SecMDGM mechanism is based on the theory of first−price auction, one of the fundamental theories for auction mechanism. The algorithms and framework in this subsection focus on steps 1 to 4 in Figure 1. We believe the model clustering for federation learning can be found in papers dedicated to the computation of federation learning.

The auction theory in this paper is based on multi−dimensional attributes, as shown in Equation (9), where *Q* is the set of bidder features. We should pay attention not only to the bidding price but also to the data quality, computing performance, and other factors in bidding. Therefore, a scoring function Score(*Q*, *p*) is needed to measure each feature, and each feature has its corresponding weight, which is mentioned in Equation (4). In other words, the auction’s winner needs to be measured in multiple dimensions to win finally.

Equation (9) indicates the *m* dimension feature the client *i* needs to submit, and *Q* denotes the feature array.

(9) $$\begin{array}{c} Q_{i}=\left(q_{1},q_{2},\cdots ,q_{m}\right) \end{array}$$

(10) $$\begin{array}{c} bid_{client\_i}\left(Encrypt_{pk_{1}}\left(Q\right),Encrypt_{pk_{2}}\left(p\right)\right)= \end{array}$$

$$bid_{client_{i}}\left(Encrypt_{pk_{1}}\left(q_{1}\right),\cdots ,Encrypt_{pk_{1}}\left(q_{m}\right),Encrypt_{pk_{2}}\left(p\right)\right)$$

Equation (10) indicates that client *i* submits the feature array *Q* and the bid price *p* to the seller, which refers to the server. Moreover, since the seller has distributed the auction rules and the public keys to the buyers separately, as known in the first step shown in Figure 1, the clients need to encrypt their submitted bid information separately.

Figure 1 displays the overall framework of the SecMDGM mechanism, and the mechanism is divided into seven steps. The last steps of model aggregation are well known, and the first steps are the mechanism design that is the focus of this paper. Thanks to its inclusion, participants can actively participate in the training of the model, while the security of the data is guaranteed. Algorithm 1 provides the pseudo−code. This section provides the flow of the SecMDGM mechanism, and the next section analyzes in detail how this mechanism is realized and proved. **Algorithm 1:** Federated Learning Security Mechanism in Round *b***Input**: Number of clients: *N*;    Number of participants in the last round: *K*;    Local multi−dimensional data;     Round *b* − 1.**Output**: Global model parameter ω(*t*):1.**for***t* = 1 to *T* **do**2.   *client*_1_ to *client_N_* ← *pk*_1_, *pk*_2_ and *Score*(*Q*, *p*) rules3.   **for** *i* = 1 to *N* **do**4.      *Q_i_* ← arg max *U* (*Q_i_*) − *c*(*Q_i_*, θ*_i_*) in Equation (17)5.      *p _i_* ←$\;c\left(Qi,\;\theta i\right)+\int_{\theta }^{\bar{\theta }}c_{\theta }\left(Q,t\right){\left(\frac{1-F\left(t\right)}{1-F\left(\theta \right)}\right)}^{N-1}dt$ in Equation (18)6.      *E*(*Qi*) ← encrypt with *pk*_1_7.      *E*(*pi*) ← encrypt with *pk*_2_8.      submit *bid*(*E*(*Q_i_*), *E*(*p_i_*))9.   **end for**10.   $\;\;\;\;\;\;\;\;\;E\left(Score\right)\leftarrow \;E_{pk_{1},\;pk_{2}}\left(U\left(q_{1},\cdots ,q_{m}\right)-p\right)$11.$\;\;\;\;\;\;\;\;\;Score\leftarrow {\;D}_{sk_{1},\;sk_{2}}(E\left(Score\right)$12.   win← 0 or 113.   Add winner to round *b* − 1 for federated learning14.   Complete the auction15.   **for** *i* = 1 to *K* + 1 **do**16.      *client_i_* ← *pk*_3_, *p _i_*, global ω(*t*) from server17.      $\omega_{i}\left(t\;+\;1\right)$ ← trains the *client_i_*18.      server ← *E_pk_*_3_(ω*_i_*(*t* + 1))19.   **end for**20.   ω(*t* + 1) ← $\omega \left(t+1\right)=\frac{\sum_{i=1}^{N}C_{i}D_{sk_{3}}\omega_{i}\left(t+1\right)}{\sum_{i=1}^{N}C_{i}}$ in Equation (8)21.**end for**

### 3.2. Cryptographic Utility and Score Functions for the SecMDGM Mechanism

The utility function is a commonly used measurement method in microeconomics, which is used to measure buyers’ satisfaction when they consume an item. In this paper, it is the satisfaction of the server with the bidding information provided by the clients. This is a subjective feeling perception, so there are many different types of utility functions and the server determines the weights of the features.

The indifference curve is used to show that items of two groups in different combinations can provide the same utility. The consumer’s choice of any point on the curve is of equal utility to the consumer because the combination represented by any point provides the consumer with no difference in satisfaction. Hence, it is termed the utility’s indifference curve. The multi−dimensional information in the bidding of clients is such a combination. We must ensure that it conforms to the federal learning requirements by giving the suitable utility function and the corresponding indifference curve.

There are two typical forms of indifferentiable curves, the curve for perfect substitutes and the curve for perfect complements.

**Figure 5:**
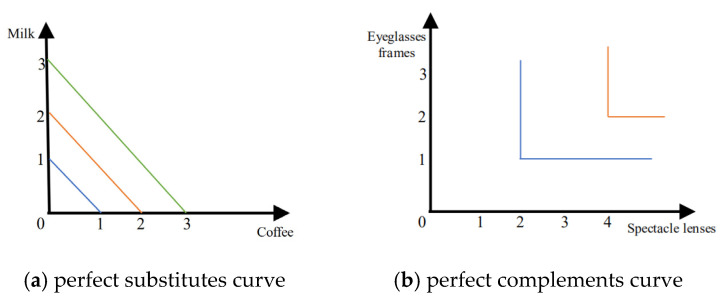
Indifference curve.

Take two items, for example. If a buyer wants to buy a thirst quencher when there are only two drinks to choose from, milk and coffee, then they are perfect substitutes, i.e., the buyer who buys milk will not buy coffee. As shown in Figure 5a, the three indifference curves represent the different demands for drinks. Equation (11) represents the utility function of the corresponding perfect substitutes.

(11) $$U\left(q_{1},q_{2}\right)=\alpha_{1}q_{1}+\alpha_{2}q_{2}$$

If a buyer wants to buy eyeglasses, then lenses and frames are perfect complementary items because they must be presented together. As shown in Figure 5b, the two indifference curves represent the different demands for pairs of eyeglasses, respectively. Equation (12) represents the utility function of the corresponding perfect complements. In addition to the different results in the calculation of the utility function, the main difference between perfect substitutes and perfect complements is the different results in the marginal rates of substitution (MRS). The MRS for perfect substitutes is 0 (Equation (13)), and the latter result is ∞.

(12) $$U\left(q_{1},q_{2}\right)=\min\left(\alpha_{1}q_{1},\alpha_{2}q_{2}\right)$$

(13) $$MRS=-\frac{\Delta q_{2}}{\Delta q_{1}}$$

The multi−dimensional features of clients, such as CPU performance, memory capacity, hard disk capacity, network bandwidth, and other performance, can replace each other, so the perfect substitution utility function is suitable for calculating the multi−dimensional information in federated learning.

**Figure 6:**
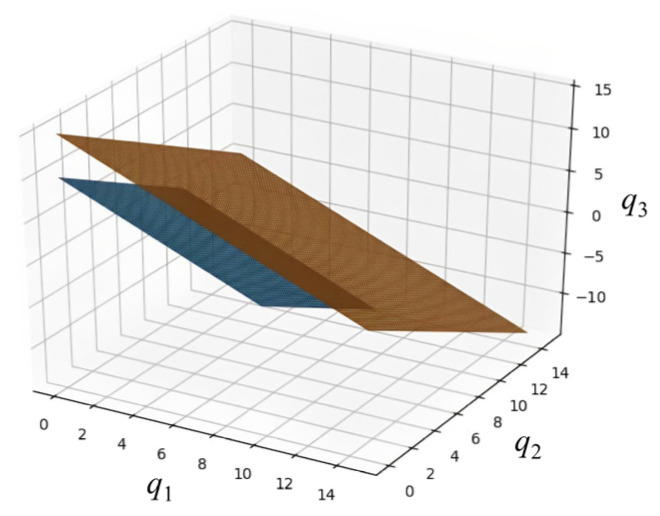
Three−dimensional indifference curve of the perfect substitution utility function.

Figure 6 represents the perfect−substitution−type preference relationship (indifference curve) for three−dimensional features when *m* is equal to three: *q_1_*, *q_2_*, and *q*. Figure 6 is an abstract description, so we do not consider the weight of each feature. We also do not need to consider the weights in the theoretical proof because they do not affect the proof of the mechanism design. The indifference curve means that for different combinations of features, all the points on the same surface have the same degree of utility for the server. This figure provides a better understanding of the utility function of the multi−dimensional auction.

The perfect substitution utility function has excellent theoretical support as a type of utility function. However, in federated learning, the training usually involves multiple rounds. In addition, the clients are generally not fixed, i.e., the auction is conducted in multiple rounds. To ensure that the multi−dimensional information of participants in different rounds is not leaked and the weights of the server are not leaked, we encrypted the perfect substitution utility function in Equation (14).

(14) $$\begin{array}{c} U\left(q_{1},\cdots ,q_{m}\right)=\alpha_{1}q_{1}+\alpha_{2}q_{2}+\cdots +\alpha_{m}q_{m} \end{array}$$

$$=D_{sk_{2}}E_{pk_{2}}\left(\alpha_{1}q_{1}\right)+E_{pk_{2}}\left(\alpha_{2}q_{2}\right)+\cdots +E_{pk_{2}}\left(\alpha_{m}q_{m}\right)$$

$$=D_{sk_{2}}E_{pk_{2}}(D_{sk_{1}}(E_{pk_{1}}\left(\alpha_{1}\right)*E_{pk_{1}}\left(q_{1}\right)$$

$$=D_{sk_{2}}E_{pk_{2}}(D_{sk_{1}}(E_{pk_{1}}\left(\alpha_{1}\right)*E_{pk_{1}}\left(q_{1}\right)+E_{pk_{2}}\left(D_{sk_{1}}\left(E_{pk_{1}}\left(\alpha_{2}\right)*E_{pk_{1}}\left(q_{2}\right)\right)\right)$$

$$+\cdots +E_{pk_{2}}\left(D_{sk_{1}}\left(E_{pk_{1}}\left(\alpha_{m}\right)*E_{pk_{1}}\left(q_{m}\right)\right)\right))$$

Equation (6) is the utility function of the security mechanism proposed in this paper. The server provides two public keys: *pk_1_* for the multiplication operation and *pk_2_* for the addition operation. Since the intermediate values after the encryption operation no longer contain the original plaintext data, their security is guaranteed. 

The score function is Equation (15), which is the utility function minus the price *p* submitted by the client when bidding. The following equation provides the encryption and decryption process of the scoring function, whose security guarantees are discussed in Section 2, so only its secure calculation process is shown here. The scoring function is used as part of the rules of the SecMDGM mechanism. Since we introduced the multi−dimensional information, a corresponding scoring function is needed to add as a supplement to the first−price auction rules.

(15) $$\begin{array}{cc} Score_{client_{i}} & =U\left(q_{i_{1}},\cdots ,q_{i_{m}}\right)-p_{i}\\ & =D_{sk_{2}}E_{pk_{2}}\left(U\left(q_{i_{1}},\cdots ,q_{i_{m}}\right)-p_{i}\right)\\ & =D_{sk_{2}}E_{pk_{2}}\left(U\left(q_{i_{1}},\cdots ,q_{i_{m}}\right)\right)-D_{sk_{2}}E_{pk_{2}}\left(p_{i}\right) \end{array}$$

The encrypted utility and scoring functions in this paper simultaneously ensure that (1) the communication process is secure when the client submits features and price to the server and (2) other malicious parties do not steal the parameters of the server when the auction is being executed. In addition, with these two functions, we developed scoring rules for multi−dimensional auctions. Section 3.3 and Section 3.4 will prove that the mechanism discussed in this paper can be guaranteed in theory due to the introduction of the scoring function.

### 3.3. Maximize the Expected Profit of the SecMDGM Mechanism 

In this section, we prove the necessity of the SecMDGM mechanism to maximize the overall profit. This necessity is reflected in two aspects:

(1) The SecMDGM mechanism in this paper is based on multi−dimensional auction theory, which is more in line with the real auction of federated learning. Not only the price but also qualities are considered.
(2) An excellent mechanism enables the server to spend less but achieves a higher performance of the model. At the same time, all the participants are willing to submit more real data and local computing resources for the federated learning task to obtain higher profits.

The first point of necessity is intuition, and the higher the dimensionality of features, the higher the quality of the results. However, because of the introduction of the first point, we need more theory to ensure that the second point holds. We can measure the second point of necessity by the scoring function in the server, which is referred to in the previous section. The profit can be calculated by Equation (16). The profit is equal to the expected revenue given by the server minus the cost of the participation of the winner. The cost *c* of the winner is related not only to the parameter *Q* but also to the privacy parameter θ.

(16)  profit(Q,p|θ)=(p−c(Q,θ))∗Prob(win|Score(Q,p))

How can we design the incentive mechanism so that the two parties of federated learning maximize their profits? Submitting the real data of *Q* and *p* will be the optimal strategy. Equations (17) and (18) are the optimal bidding strategies. The proof is given below.

**Theorem** **1.** *Maximize the Expected Profit from the SecMDGM Mechanism*.

(17) $$\begin{array}{c} Q_{winner}=argmaxU\left(Q\right)-c\left(Q,\theta \right) \end{array}\;$$

(18) $$\begin{array}{c} p_{winner}=c\left(Q_{winner},\theta \right)+\int_{\theta }^{\bar{\theta }}c_{\theta }\left(Q,t\right){\left(\frac{1-F\left(t\right)}{1-F\left(\theta \right)}\right)}^{N-1}dt \end{array}\;$$

**Proof of Theorem** **1.** We use the reduction to absurdity to prove Equation (17).

Suppose that there is a dominant bidding strategy bid(Q,p) in client *i*, who get the maximize maximum expected profit.

Contradiction: Exists another bidding strategy $bid\left(Q^{\prime },p^{\prime }\right)\;\neq bid\left(Q,p\right)$, satisfies the following equations.

(19) $$\{\begin{array}{c} Q^{\prime }=argmaxU\left(Q^{\prime }\right)-c\left(Q^{\prime },\theta^{\prime }\right)\\ p^{\prime }=p+U\left(Q^{\prime }\right)-U\left(Q\right) \end{array}$$

If the following conditions are satisfied, then the strict predominance of bid(Q,p) is replaced by $bid\left(Q^{\prime },p^{\prime }\right)$.

(20) $$profit(Q^{\prime },p^{\prime }|\theta \prime )\geq \;profit(Q,p|\theta )$$

Transformation of Equation $p^{\prime }=p+U\left(Q^{\prime }\right)-U\left(Q\right)$ can gets $U\left(Q\right)-p=U\left(Q^{\prime }\right)-p^{\prime }$ and $Score\left(Q,p\right)=Score\left(Q^{\prime },p^{\prime }\right)$. Since the scores are equal, then $Q^{\prime }=Q=Q_{winner}$**.** This contradicts with $bid\left(Q^{\prime },p^{\prime }\right)\;\neq bid\left(Q,p\right)$. Therefore, $Q^{\prime }=argmaxU\left(Q\prime \right)-c\left(Q\prime ,\theta \prime \right)\;holds\;$ and Equation (17) holds.

Equivalence Proof Equation (16): From the above, it is clear that once Equation (20) is proved then Equation (17) will hold.

(21) $$profit(Q^{\prime },p^{\prime }|\theta^{\prime })=\left(p^{\prime }-c\left(Q^{\prime },\theta^{\prime }\right)\right)*Prob(win|Score\left(Q^{\prime },p^{\prime }\right))$$

$$=(p-c\left(Q,\theta \right)+\left(U\left(Q^{\prime }\right)-c\left(Q^{\prime },\theta^{\prime }\right)-\left(\left(U\left(Q\right)-c\left(Q,\theta \right)\right)\right)\right)\;*Prob(win|Score\left(Q^{\prime },p^{\prime }\right))$$

$$\geq \left(p-c\left(Q,\theta \right)\right)*Prob(win|Score\left(Q^{\prime },p^{\prime }\right))$$

=profit(Q,p|θ)

Therefore, Equation (20) holds, contradiction exists and Equation (17) holds.

The proof of Equation (18) is described in detail in articles [43,44], where *F*() is the distribution function. Due to space limitations, it will not be repeated in this paper. Since the values of *Q* and *p* are the client’s own strategy actions that are carried out locally before bidding, encryption is not required at this step. Therefore, the SecMDGM mechanism has the optimal strategies if the participants join the game. *□*

### 3.4. Pareto Optimal Mechanism and Incentive Compatibility (IC) of the SecMDGM Mechanism

Section 3.3 proved that the game has optimal strategies under the rules set by the SecMDGM mechanism, but it does not mean that the mechanism itself is optimal. In this section, we prove that the mechanism itself is optimal.

It is generally believed that the essential criteria to evaluate a mechanism include the effective allocation of resources and maximization of profit. Whereas efficient resource allocation needs to be demonstrated by proving Pareto optimality, maximizing profit must consider incentive compatibility. Therefore, we prove that the SecMDGM mechanism satisfies Pareto optimality and incentive compatibility.

**Theorem** **2.** *Pareto Optimal of the SecMDGM Mechanism.*

The Pareto optimal mechanism can maximize the social surplus. It describes a state of optimal allocation of resources. There is no way to make it more profitable without causing losses to the other party.

**Proof of Theorem ** **2.** Equation is: Surplus=U(Q)−c(q). Since Equation (17) has proved that in the optimal strategy, $Q_{winner}=argmaxU\left(Q\right)-c\left(Q,\theta \right)$.

These two equations are understood from different perspectives, but their results are the same, and we differ in obtaining the value of c from a global perspective, but since the mechanism proves Equation (17). The optimal strategy of the participants is to make the whole mechanism Pareto optimal. This means Surplus can be the maximum when $Q=Q_{winner}$. Pareto Optimal of the SecMDGM Mechanism holds. □

**Theorem** **3.** *Incentive Compatibility (IC) of the SecMDGM Mechanism.*

The federated learning mechanism allows clients to pursue their individual profits while maximizing collective profit. That means incentive compatible.

**Proof of Theorem** **3.** If there exist clients who bid $bid\left(Q^{\prime },p\right)$, where $\exists j,q_{j}>q_{j^{\prime }}$. Equation (17) has proved that when $Q_{winner}=argmaxU\left(Q\right)-c\left(Q,\theta \right)$, the client can maximize the expected profit. From the utility function, it is known that $U\left(Q\right)>U\left(Q^{\prime }\right)$, and it is deduced that $Score\left(Q\right)>Score\left(Q^{\prime }\right)$. Thus, the winner that the mechanism chooses is the one that pursues their individual interests. □

The above two theorems show that the SecMDGM mechanism discussed in this paper can maximize the profit of both client and server and the client can actively submit more data and other multi−dimensional parameters, thus maximizing personal profit.

## 4. Experimental Verification of Federated Learning Security Mechanisms

To verify that the SecMDGM mechanism is not only secure and necessary but also effective, we simulate the federated learning process of the auction through experiments.

It is well known that algorithmic game theory can be divided into game strategy solving and mechanism design. Section 3.2 proves that the SecMDGM mechanism has the optimal game strategy. However, we focus on exploring the design of the mechanism so the experiments do not pursue the optimization of the game strategy but verify the effectiveness of the SecMDGM mechanism when the optimal solution has been proven.

Therefore, the weights of the score function are arbitrary and values will be given below. Suppose you want to reproduce the verification experiments to meet the rules of the SecMDGM mechanism. In that case, you can obtain some performance improvements of federated learning by using arbitrary values. Moreover, the features sought by each server are different, so the weight value is not essential.

In addition, no existing federal learning work can implement security mechanisms and multi−dimensional auctions. Therefore, the benchmark for this experiment is federal learning computing without a mechanism design in the same dataset.

To reduce the influence of the machine model on the results, the servers of this experiment are all based on the same operating system (Cents 7.9), which has the same performance as the Alibaba Cloud Server, with the same CPU model: Intel Xeon E5−2682 v4. The number of cores and memory capacity are taken as variables.

### 4.1. Data and Environment

The model trained for the experiments is a logistic regression model, with a public dataset involving breast cancer, with 569 participants and 30 dimensions. The features are calculated from the digital image of a fine needle aspiration (FNA) of a breast mass. They describe the characteristics of the nucleus existing in the image. Table 1 provides a classification of this dataset.

As the multi−dimensional auction is perfectly consistent with the vertical data, this experiment is based on federal learning of vertical data and the features of the breast cancer dataset meet this requirement. As mentioned in the previous section, $Q=q_{1}\cdots q_{m}$ denotes the multi−dimensional features submitted by clients. It can be CPU performance, memory capacity, number of local data users, number of local data features, etc.

In this experiment, we select two−dimensional features $Q=q_{1},q_{2}$ for federated learning auctions and arbitrarily select $a_{1}=0.4,a_{2}=0.6$. As federated learning is usually a multi−round process, the multi−dimensional information of the client is important and should be encrypted. The security of encryption and decryption, as well as the steps in the auction mechanism, have been discussed in detail in the preceding sections. This section deliberately does not emphasize the encryption and decryption process but is completed under ciphertext.

To facilitate an intuitive presentation of the effectiveness of the SecMDGM mechanism in federated learning, the experiments began with round *b* of bidding. In round *b* − 1, the accuracy of the logistic regression model was set to about 90% to reflect the improvement in the performance of different participants. In this experiment, *q*_1_ denotes the performance of the client’s computer, *q*_2_ represents the number of features of local data, and *p* represents the bid price. For the three features, we perform Min–Max normalization, which is shown in Equation (22), and complete the auction process in steps according to the specified parameter weights and auction rules of the SecMDGM mechanism.

(22) $$q^{nor}=\frac{q-q^{min}}{q^{max}-q^{min}}$$

As shown in Table 2, after normalization, encryption, and decryption, using the perfect substitution utility and score function in Section 3, the highest scorer is the winner of this round of the first−price auction. Meanwhile, the winner needs to pay RMB 300 and provide 1VCPU 1Gib and 15−dimensional local data to participate in the model training. Therefore, client three can add to the training of the federated learning model.

**Figure 7:**
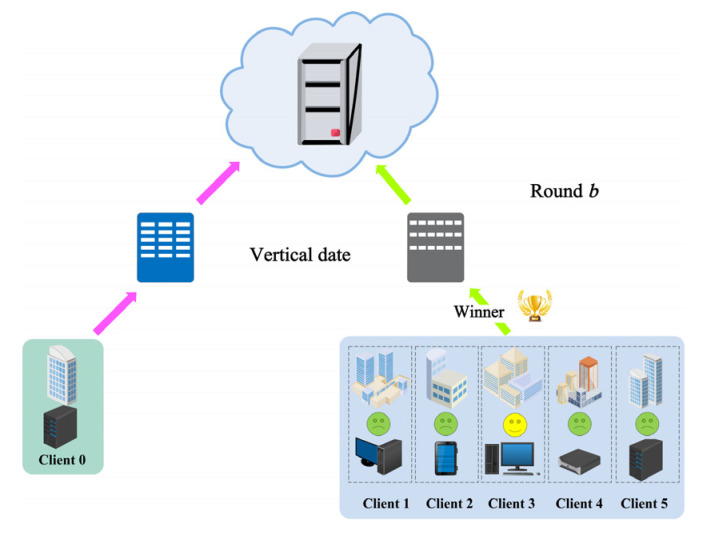
Two−dimensional auction in federated learning with vertical data.

The process of aggregation model of federated learning is also under encryption. To present the experimental results, the encryption and decryption processes are not emphasized in the experimental process. Figure 7 shows the flowchart of round *b* bidding, with five bidders and one winner. It is a partial diagram of Figure 1, which provides us with a more visual understanding of the local process of the experiment. Although the weights are chosen arbitrarily, the experimental data are from public datasets and the complete auction rules, cryptographic functions, and computational functions are provided. Thus, this is a reproducible experiment.

### 4.2. Results and Analysis

Section 3 proves the security provided by and the necessity of the SecMDGM mechanism based on multi−dimensional auctions. This section corroborates that the SecMDGM mechanism can improve the model’s accuracy by providing effective incentive to the five participants. Table 3 compares the work in this paper with state−of−the−art algorithms. We are the first to implement both the cryptographic mechanism and multi−dimensional auction in federated learning under vertical data. This is why we did not reproduce them in the experiments. On the basis of the baseline, we compare the winner and a random client under the SecMDGM mechanism.

There are many different scenarios of federated learning. However, what remains limited is the budget funds and the number of participants on the server. The aim of the SecMDGM mechanism is to achieve a better model with less budget overhead, and we conduct model training on federated learning based on vertical data. The SecMDGM mechanism enables each client to participate actively. This can ensure a better model for the server and better profits for the client, and the multi−dimensional privacy features will not be leaked to other participants.

The baseline of this paper is the model in round *b* − 1. To make the gap in the accuracy more apparent, we adjust the accuracy of the baseline to 90%. The experiments are compared by comparing random clients with the winner under the auction mechanism in round *b*, which can highlight the effectiveness of the SecMDGM mechanism.

**Figure 8:**
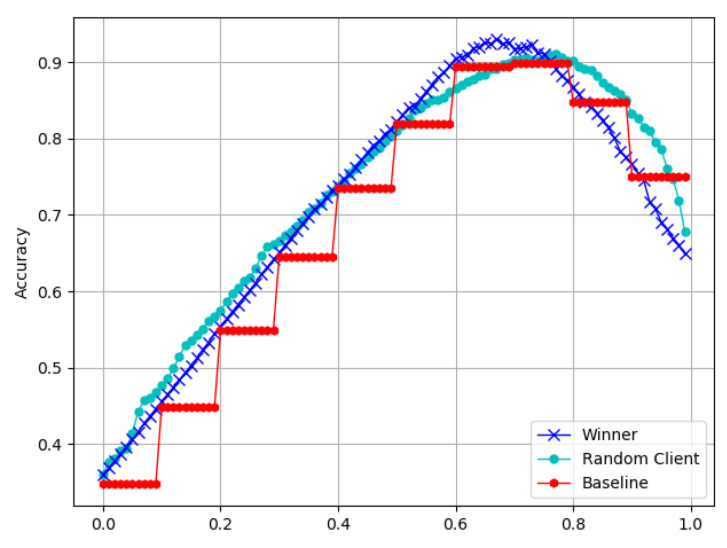
Accuracy for different participants.

Figure 8 shows the accuracy curves of the models with different participants. The accuracy of the models in round b is better than that of the models in round *b −* 1, and the accuracy of the model for the winner is obviously better than that for a random client. This can highlight the effectiveness of the SecMDGM mechanism.

**Figure 9:**
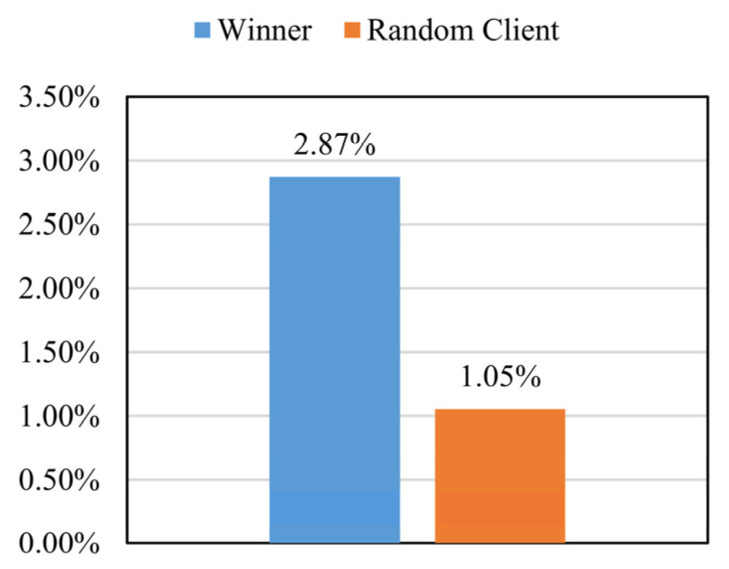
Increased accuracy of different participants.

More precisely, Figure 9 displays the improvement in the model accuracy by two different mechanisms. The SecMDGM mechanism is found to improve the model accuracy by nearly 3 times compared to that using the method of random clients. The optimal mechanism showed a more significant improvement than the random auction mechanism. The performance of the randomly selected client improved by about 1%, with almost no change. In contrast, for the aggregation model based on the vertical data, the performance of the winner improved by 2.73 times.

**Figure 10:**
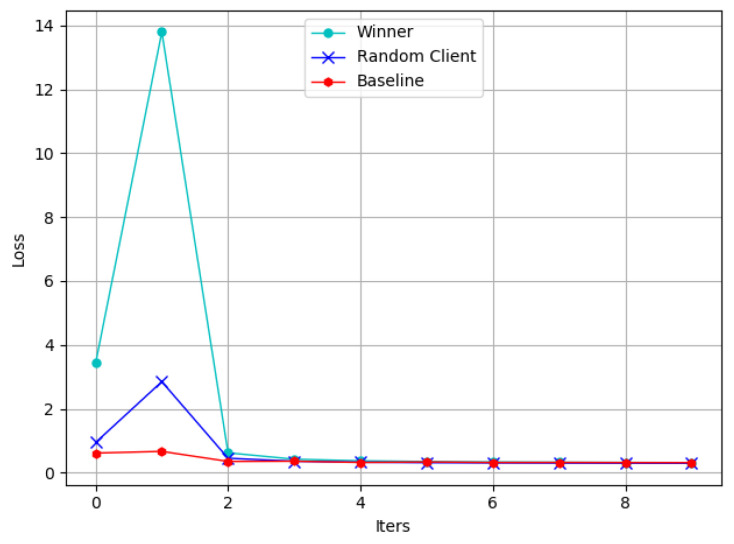
Loss for different participants.

Figure 10 shows the loss curves of different models. Due to more characteristic dimensions of the winner, its loss curve shows a more significant jump than others in the first few iterations. Moreover, it can converge quickly within a reasonable range, which is acceptable and reasonable.

**Figure 11:**
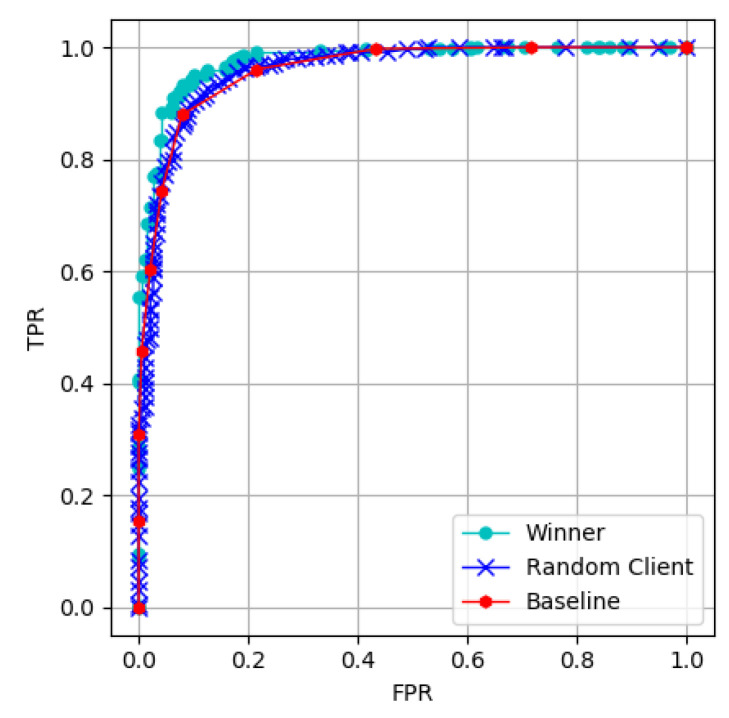
Graph of the ROC and the AUC.

The receiver operating characteristic (ROC) and the area under the ROC curve (AUC) are often used to measure the goodness of the model. The horizontal axis of the ROC curve is the FPR, and the vertical axis is the TPR. The larger the AUC, the better the model. Figure 11 shows that the winner model is better than the randomly selected client.

As the experiment in this section only verifies the effectiveness of the design of the SecMDGM mechanism, the values of *m* in *p*_1_~*p_m_* and *a*_1_~*a_m_* are far lower than in the real scenes. Nevertheless, they are sufficient to reflect the effectiveness of the SecMDGM mechanism.

The SecMDGM mechanism in federated learning is theoretically proved in Section 2 and Section 3 and verified by experiments in Section 4. Therefore, the multi−dimensional secure federated learning mechanism in this paper is secure, necessary, safe, and effective. In future work, we can not only consider the mechanism but also discuss the optimal strategy of the game. We believe that this will lead to improved results.

## 5. Conclusions

There is no doubt about the importance of federated learning, and research has gradually focused on the security of the aggregation model. However, the issue is not only in the computation but also in motivating participants to join in the federated learning. In this paper, first, the multi−dimensional auction mechanism has been verified theoretically and experimentally to improve the performance of the model. However, federated learning participants often change, and the provided data and computational performance will dynamically change for fear of leaks. Second, security is considered to make multi−dimensional auctions more practical, and we solve this challenge by the partial homomorphic encryption scheme. Through theoretical proof and experimental verification, the security, necessity, and effectiveness of the SecMDGM mechanism are proved. However, the actual environment is more complex and changeable, and more difficult problems remain to be solved, such as multi−winner auctions and multi−dimensional incentive mechanisms with budget constraints, which need to be discussed in the future.

## Figures and Tables

**Table 1:** Dataset of breast cancer *.

Classification	Number
Benign	357
Malignant	212

* Retrieved from https://archive.ics.uci.edu/ml/datasets/Breast+Cancer+Wisconsin+%28Diagnostic%29, 29 November 2022.

**Table 2:** The *bth* bid for federated learning.

Client	*q*_1_	*q_2_*	*p*	Score	Win
1	1vCPU 1Gib	9 features	RMB 400	−0.21	0
2	2vCPU 8Gib	5 features	RMB 600	−0.18	0
**3**	**1vCPU 1Gib**	**15 features**	**RMB 300**	**0.04**	**1**
4	1vCPU 4Gib	10 features	RMB 350	−0.01	0
5	1vCPU 4Gib	5 features	RMB 400	0.34	0

**Table 3:** Comparison with state−of−the−art algorithms.

	Mechanism Design	Encrypted Mechanism	Federated Learning	Encrypted Federated Learning	Vertical Data
Federated Avering [45]			√		
Pysyft [46,47]			√	√	
FMore [48]	√		√		
FATE [49]			√	√	√
**This Paper**	**√**	**√**	**√**	**√**	**√**

## Data Availability

The data used to support the funding of this study are included within the article.

## References

[1] Yu K., Lin L., Alazab M., Tan L., Gu B. (2020). Deep learning−based traffic safety solution for a mixture of autonomous and manual vehicles in a 5g−enabled intelligent transportation system. IEEE Trans. Intell. Transp. Syst..

[2] Alazab M., Macfarlane K. Why Telegram Became the Go−To App for Ukrainians–Despite Being Rife with Russian Disinformation. The Conversation 2022.

[3] Sun J., Xu G., Zhang T., Alazab M., Deng R.H. (2022). A practical fog−based privacy−preserving online car−hailing service system. IEEE Trans. Inf. Forensics Secur..

[4] Ebrahimpour G., Haghighi M.S., Alazab M. (2022). Can blockchain be trusted in industry 4.0? study of a novel misleading attack on bitcoin. IEEE Trans. Ind. Inform..

[5] Yuan J., Jiang Q., Pan Y. (2022). The influence mechanism of knowledge network allocation mechanism on knowledge distillation of high−tech enterprises. Comput. Intell. Neurosci..

[6] Maddikunta P.K.R., Pham Q. (2022). −V.; Prabadevi, B.; Deepa, N.; Dev, K.; Gadekallu, T.R.; Ruby, R.; Liyanage, M. Industry 5.0: A survey on enabling technologies and potential applications. J. Ind. Inf. Integr..

[7] Kattepur A., Khemkha S. (2022). Aspects of mechanism design for industry 4.0 multi−robot task auctioning. EAI Endorsed Trans. Smart Cities.

[8] Raphael K., Balaguer J., Tacchetti A., Weinstein A., Zhu T., Hauser O., Williams D., Campbell−Gillingham L., Thacker P., Botvinick M. (2022). Human−centered mechanism design with democratic ai. Nat. Hum. Behav..

[9] Voigt P., Von dem Bussche A. (2017). The EU General Data Protection Regulation (GDPR). A Practical Guide.

[10] Thakur P.S., Kiran U., Sahu O.P. (2022). A Review on: Machine Learning Techniques to Mitigate Security Risks in IOT Framework State of the Art in Futuristic Communication and Network Technologies.

[11] Yang Q., Liu Y., Chen T., Tong Y. (2019). Federated machine learning: Concept and applications. ACM Trans. Intell. Syst. Technol. (TIST).

[12] Du Z., Wu C., Yoshinaga T., Yau K.-L.A., Ji Y., Li J. (2020). Federated learning for vehicular internet of things: Recent advances and open issues. IEEE Open J. Comput. Soc..

[13] Sim R.H.L., Zhang Y., Chan M.C., Low B.K.H. Collaborative machine learning with incentive−aware model rewards. Proceedings of the International Conference on Machine Learning.

[14] Sarikaya Y., Ercetin O. (2019). Motivating workers in federated learning: A stackelberg game perspective. IEEE Netw. Lett..

[15] Konecny J., McMahan B., Ramage D. (2015). Federated optimization: Distributed optimization beyond the datacenter. arXiv.

[16] Alazab M., Rm S.P., Parimala M., Maddikunta P.K.R., Gadekallu T.R., Pham Q. (2021). −V. Federated learning for cybersecurity: Concepts, challenges, and future directions. IEEE Trans. Ind. Inform..

[17] Kandati D.R., Gadekallu T.R. (2022). Genetic clustered federated learning for covid−19 detection. Electronics.

[18] Supriya Y., Tech M., Bindu G.H., Tech M., Reddy K.D. (2018). Securing cloud data using identity−based encryption scheme under key exposure. Int. J. Sci. Res. Comput. Sci. Eng. Inf. Technol..

[19] Javed A.R., Rehman S.U., Khan M.U., Alazab M., Reddy T. (2021). Canintelliids: Detecting in−vehicle intrusion attacks on a controller area network using cnn and attention−based Gru. IEEE Trans. Netw. Sci. Eng..

[20] Zhan Y., Zhang J., Hong Z., Wu L., Li P., Guo S. (2021). A survey of incentive mechanism design for federated learning. IEEE Trans. Emerg. Top. Comput..

[21] Wang J., Zhao L., Liu J., Kato N. (2019). Smart resource allocation for mobile edge computing: A deep reinforcement learning approach. IEEE Trans. Emerg. Top. Comput..

[22] Von Neumann J., Morgenstern O. (2007). Theory of Games and Economic Behavior.

[23] Roughgarden T. (2010). Algorithmic game theory. Commun. ACM.

[24] Myerson R.B. (1989). Mechanism design. Allocation, Information and Markets.

[25] Börgers T., Krahmer D. (2015). An Introduction to the Theory of Mechanism Design.

[26] Nisan N., Ronen A. (2001). Algorithmic mechanism design. Games Econ. Behav..

[27] Yang P., Yan S., Zhu D., Wang J., Wu F., Yan Z., Yan S. (2022). Improved sparrow algorithm based on game predatory mechanism and suicide mechanism. Comput. Intell. Neurosci..

[28] Murali M., Thyagarajan K., Reddy K.D. (2014). Document clustering for digital devices: An approach to improve forensic analysis. Int. J. Eng. Res. Manag. Technol..

[29] Vickrey W. (1961). Counterspeculation, auctions, and competitive sealed tenders. J. Financ..

[30] Shen W., Peng B., Liu H., Zhang M., Qian R., Hong Y., Guo Z., Ding Z., Lu P., Tang P. (2020). Reinforcement mechanism design: With applications to dynamic pricing in sponsored search auctions. AAAI Conf. Artif. Intell..

[31] Choi H., Mela C.F., Balseiro S.R., Leary A. (2020). Online display advertising markets: A literature review and future directions. Inf. Syst. Res..

[32] Lebrun B. (1996). Existence of an equilibrium in first price auctions. Econ. Theory.

[33] Ion M., Kreuter B., Nergiz A.E., Patel S., Saxena S., Seth K., Raykova M., Shanahan D., Yung M. On deploying secure computing: Private intersection−sum−with−cardinality. Proceedings of the 2020 IEEE European Symposium on Security and Privacy (EuroS&P).

[34] Walker A., Patel S., Yung M. (2019). Helping Organizations Do More without Collecting More Data. Google Security Blog. https://security.googleblog.com/2019/06/helping-organizations-do-more-without-collecting-more-data.html.

[35] McAfee R.P., McMillan J. (1987). Auctions and bidding. J. Econ. Lit..

[36] Laffont J. (1995). −J.; Ossard, H.; Vuong, Q. Econometrics of first−price auctions. Econom. J. Econom. Soc..

[37] Grundl S., Zhu Y. Robust Inference in First−Price Auctions: Overbidding as an Identifying Restriction. Finance and Economics Discussion 2019, 1–51. https://www.sciencedirect.com/science/article/abs/pii/S0304407622001221.

[38] Bichler M. (2000). An experimental analysis of multi−attribute auctions. Decis. Support Syst..

[39] Acar A., Aksu H., Uluagac A.S., Conti M. (2018). A survey on homomorphic encryption schemes: Theory and implementation. ACM Comput. Surv..

[40] Mahajan P., Sachdeva A. (2013). A study of encryption algorithms AES, des and RSA for security. Glob. J. Comput. Sci. Technol..

[41] Paillier P. (1999). Public−key cryptosystems based on composite degree residuosity classes. Proceedings of the International Conference on the Theory and Applications of Cryptographic Techniques.

[42] Hard A., Rao K., Mathews R., Ramaswamy S., Beaufays F., Augenstein S., Eichner H., Kiddon C., Ramage D. (2018). Federated learning for mobile keyboard prediction. arXiv.

[43] Che Y.-K. (1993). Design competition through multidimensional auctions. RAND J. Econ..

[44] Riley J.G., Samuelson W.F. (1981). Optimal auctions. Am. Econ. Rev..

[45] McMahan B., Moore E., Ramage D., Hampson S., Arcas B.A.Y. (2017). Communication−efficient learning of deep networks from decentralized data. Artificial Intelligence and Statistics.

[46] Ryffel T., Pointcheval D., Bach F., Dufour−Sans E., Gay R. (2019). Partially encrypted deep learning using functional encryption. Adv. Neural Inf. Process. Syst..

[47] Ryffel T., Trask A., Dahl M., Wagner B., Mancuso J., Rueckert D., Passerat−Palmbach J. (2018). A generic framework for privacy preserving deep learning. arXiv.

[48] Zeng R., Zhang S., Wang J., Chu X. Fmore: An incentive scheme of multi−dimensional auction for federated learning in MEC. Proceedings of the 2020 IEEE 40th International Conference on Distributed Computing Systems (ICDCS).

[49] Yang Q., Liu Y., Cheng Y., Kang Y., Chen T., Yu H. (2019). Federated learning. Synth. Lect. Artif. Intell. Mach. Learn..

